# An investigation of the neural substrates of mind wandering induced by viewing traditional Chinese landscape paintings

**DOI:** 10.3389/fnhum.2014.01018

**Published:** 2015-01-06

**Authors:** Tingting Wang, Lei Mo, Oshin Vartanian, Jonathan S. Cant, Gerald Cupchik

**Affiliations:** ^1^Research Center for Psychology and Special Education, National Institute of Education SciencesBeijing, China; ^2^Department of Psychology, South China Normal UniversityGuangzhou, China; ^3^Department of Psychology, University of Toronto ScarboroughToronto, ON, Canada

**Keywords:** Chinese painting, relaxation, flanker task, cognitive control, attention

## Abstract

The present study was conducted to investigate whether the calming effect induced by viewing traditional Chinese landscape paintings would make disengagement from that mental state more difficult, as measured by performance on a cognitive control task. In Experiment 1 we examined the subjective experience of viewing traditional Chinese landscape paintings vs. realistic oil landscape paintings in a behavioral study. Our results confirmed that, as predicted, traditional Chinese landscape paintings induce greater levels of relaxation and mind wandering and lower levels of object-oriented absorption and recognition, compared to realistic oil landscape paintings. In Experiment 2 we used functional Magnetic Resonance Imaging to explore the behavioral and neural effects of viewing traditional Chinese landscape paintings on a task requiring cognitive control (i.e., the flanker task)—administered *immediately* following exposure to paintings. Contrary to our prediction, the behavioral data demonstrated that compared to realistic oil landscape paintings, exposure to traditional Chinese landscape paintings had no effect on performance on the flanker task. However, the neural data demonstrated an interaction effect such that there was greater activation in the inferior parietal cortex and the superior frontal gyrus on incongruent compared with congruent flanker trials when participants switched from viewing traditional Chinese landscape paintings to the flanker task than when they switched from realistic oil landscape paintings. These results suggest that switching from traditional Chinese landscape paintings placed greater demands on the brain’s attention and working memory networks during the flanker task than did switching from realistic oil landscape paintings.

## INTRODUCTION

Traditional Chinese landscape painting is one of the oldest continuous artistic traditions in the world. It involves essentially the same techniques as calligraphy and is done with a brush dipped in black or colored ink, often referred to as *shuimohua* (water and ink). Among all content types, landscape is the most popular motif in traditional Chinese painting (Zong, 2007; Law, 2011). Unlike realistic oil landscape painting, its purpose is not to reproduce exactly the appearance of nature but, rather, to embody an emotion or atmosphere that expresses its “rhythm.” That is, it conveys the experience of “being in nature” rather than “seeing nature” (Law, 2011; Stokstad and Cothren, 2011). Traditional Chinese artists put great emphasis on the spiritual qualities of the painting and its ability to reveal the inner harmony of mankind and nature, as perceived according to Taoist and Buddhist concepts (Legge, 1891; Sullivan, 1999; Zong, 2007; Chan, 2008).

Compared with realistic oil landscape paintings, which usually involve rich and bright colors, well-defined forms, and are filled with narrative details consistent with a Western tradition, traditional Chinese landscape paintings have three distinctive features in drawing style. First, the concept of “drawing-blank” (referred to as “*feibai*” in Chinese) is very important for both artists and appreciators. “Drawing-blank” is a method in traditional Chinese painting of applying pressure to the brush, which causes the hair of the brush to separate, leaving streaks of white spaces (Cahill, 1960; Zong, 2007). The empty space in traditional Chinese painting is used to give balance, to harmonize the messages in the painting, and to give prominence to concrete images (Cahill, 1960; Zong, 2007). “Drawing-blank” provides an empty space for appreciators to wander, imagine, and create (Cahill, 1960; Zong, 2007). Second, the boundaries in traditional Chinese landscape paintings are more blurred than most realistic oil landscape paintings (Cahill, 1960; Zong, 2007). That is, the edge of an object is painted in an ill-defined way so that it disappears or fades into another object or into the background (Cahill, 1960; Zong, 2007). This is similar to soft-edge paintings. According to Wolﬄin (1950), soft-edge paintings involve blurred boundary and ill-defined forms; whereas hard-edge paintings involve well-defined forms and richly narrative details. Third, compared with realistic oil landscape paintings, traditional Chinese landscape paintings do not usually contain many bright colors (Cahill, 1960; Zong, 2007). The two separate styles of traditional Chinese landscape paintings are “blue-and-green landscapes” and “ink-and-wash landscape” (Cahill, 1960; Zong, 2007). The blue-and-green landscape usually used three colors: blue, green and red pigments, to create a richly decorative style (Cahill, 1960; Zong, 2007). The ink-and-wash landscape technique relies on vivid brushwork and varying degrees of intensity of ink to express the artist’s conception of nature (Cahill, 1960; Zong, 2007). Because of the aforementioned characteristics, traditional Chinese landscape paintings engage appreciators in an attempt to construct a mental image of a landscape spontaneously. According to previous studies, soft-edge paintings like traditional Chinese landscape paintings can facilitate flexible visuospatial exploration (Berlyne and Ogilvie, 1974; Cupchik, 1974, 1976). Whereas viewers can clearly understand the contents of paintings when viewing hard-edge paintings (i.e., externally oriented cognition), viewing soft-edge paintings requires viewers to engage more to construct the mental imagery by themselves (i.e., internally oriented cognition), thereby linking the esthetic appreciation with their own experience (Cupchik et al., 2009). Thus, compared with realistic oil landscape paintings, viewing traditional Chinese landscape paintings would evoke greater levels of relaxation and mind wandering. During the appreciation, viewers usually experience relaxation and mental travel, and tend to lose themselves in the painting, which in turn induces a subjective calming “after-effect” phenomenon in the form of difficulty to disengage from the painting (Gibson, 1933). Although the effect of traditional Chinese landscape paintings on cognitive control has been recognized phenomenologically and theoretically (Cahill, 1960; Zong, 2007; Law, 2011), to date no empirical evidence for its neural bases has been provided.

With the development of the field of neuroesthetics, researchers have begun to uncover the neural bases of arts appreciation. A variety of studies using painting, music, dance, sculpture, and architecture have shown that the perception of “beauty” is reliably correlated with activity of cortical and subcortical areas implicated in the processing of reward, including the orbitofrontal cortex (OFC) and the striatum (Blood et al., 1999; Blood and Zatorre, 2001; Brown et al., 2004; Cela-Conde et al., 2004; Kawabata and Zeki, 2004; Vartanian and Goel, 2004; Menon and Levitin, 2005; Calvo-Merino et al., 2008; Cupchik et al., 2009; Kirk et al., 2009; Koelsch, 2010; Di Dio et al., 2011; Ishizu and Zeki, 2011; Vessel et al., 2012). Importantly, esthetic appreciation appears to emerge as a product of the flexible interplay between the top–down orientation adopted by the viewer and the bottom–up features facilitated by the stimuli, involving an interaction between their respective cognitive and affective neural systems (Nadal et al., 2008; Cupchik et al., 2009).

However, previous research has mainly concentrated on the esthetic attributes of artworks. In contrast, little attention has been paid to the effect of artworks on cognitive processing as measured by relevant tasks, and the neural mechanisms that underlie this process. We propose that, compared with viewing realistic oil landscape paintings, the appreciation of traditional Chinese landscape paintings will be accompanied by greater difficulty in shifting attention to a different and more cognitively demanding task, especially if the subsequent task itself requires focused attention. In turn, such shifting will necessitate a higher level of cognitive control (Gibson, 1933) compared with realistic oil landscape paintings. This line of reasoning is based on the idea that the relaxation and mind wandering experienced while viewing traditional Chinese landscape paintings will make it more effortful to refocus attention on a task requiring externally oriented cognition. This effort in switching would in turn be accompanied by neural activation in areas of the brain known to underlie cognitive control in the service of task switching, primarily the prefrontal cortex (PFC) and the inferior parietal cortex (IPC; Casey et al., 2000; Hazeltine et al., 2000; Durston et al., 2003; Ridderinkhof et al., 2004; Zhu et al., 2010).

In order to investigate the effect of viewing traditional Chinese landscape paintings on cognitive control, two experiments were performed. We conducted Experiment 1 to verify that there were indeed differences in the subjective experiences of viewing traditional Chinese landscape paintings compared with realistic oil landscape paintings, which would serve to validate our stimulus set for use in the fMRI paradigm of Experiment 2. Realistic oil landscape paintings were chosen as a comparison. We hypothesized that, compared with realistic oil landscape paintings, viewing traditional Chinese landscape paintings would evoke greater levels of relaxation and mind wandering and lower levels of object-oriented immersion and recognition. This prediction is based on the well-known distinction between internally oriented vs. externally oriented cognition in relation to esthetic phenomena (Cupchik et al., 2009).

In Experiment 2, we used an event-related fMRI design to explore the behavioral and neural effects of viewing artworks on cognitive control using the flanker task (see Method). Briefly, on each trial, participants were instructed to first view a landscape painting (either traditional Chinese, realistic oil, or scrambled/control) and then *immediately* afterward (0.5 s) perform a flanker task. During the viewing of each painting, participants were asked to make a like/dislike judgment. This preference task was conducted to explicitly instill an esthetic viewing orientation (Cupchik et al., 2009) in the participant prior to engagement with the flanker task. The flanker task is a cognitive interference task and requires highly focused attention. According to previous studies, performance on the flanker task is associated with cognitive processes such as visual attention, visual perception, working memory (WM), and especially conflict resolution and cognitive control (Eriksen and Eriksen, 1974; Casey et al., 2000; Hazeltine et al., 2000; Bunge et al., 2002; Durston et al., 2003; Ridderinkhof et al., 2004; Zhu et al., 2010). During the task, participants are asked to judge the orientation of a central arrow, which is surrounded by either congruent flankers (i.e., the central arrow and the flankers are presented in the same direction, e.g., >>>>> or <<<<<) or incongruent flankers (i.e., the central arrow and the flankers are presented in different directions, e.g., >><>> or <<><<; Eriksen and Eriksen, 1974). Previous studies have found that, compared with the congruent condition, the incongruent condition is associated with lower accuracy and longer response times, and that it activates brain areas related to cognitive control such as the IPC and the PFC (Casey et al., 2000; Hazeltine et al., 2000; Bunge et al., 2002; Durston et al., 2003; Ridderinkhof et al., 2004; Zhu et al., 2010). Importantly, by purposefully minimizing the time between the picture viewing and flanker tasks (i.e., 0.5 s), we explicitly aimed to incorporate the painting viewing window into the design, thereby capturing the hemodynamic response function (HRF) associated with the flanker task *as it was being affected by immediate prior exposure to the paintings*. If it is indeed the case that viewing traditional Chinese landscape paintings will induce more mind wandering, it may make it more difficult to switch from this mental state when faced with the more cognitively demanding flanker task, which would translate into slower RTs or lower accuracy rates on incongruent compared with congruent trials. At the neural level, we predicted an interaction effect such that brain areas related to cognitive control (e.g., the IPC and the PFC) would show higher activation on incongruent trials (compared with congruent trials) after viewing traditional Chinese landscape paintings (compared with realistic oil landscape paintings). We focused our analyses on these regions because both have been reliably associated with cognitive control (IPC: Casey et al., 2000; Hazeltine et al., 2000; Durston et al., 2003; within PFC, the superior frontal gyrus (SFG), or SFG, is heavily involved in cognitive control: Casey et al., 2000; Durston et al., 2003; Ridderinkhof et al., 2004; Zhu et al., 2010).

## EXPERIMENT 1: SUBJECTIVE REPORT PARADIGM

In Experiment 1, we examined relevant aspects of the subjective experience of viewing traditional Chinese landscape paintings and realistic oil landscape paintings.

### METHOD

#### Participants

Twenty-eight (20 female, 8 male) right-handed, Chinese college-aged participants from South China Normal University in Guangzhou, China were recruited and paid for their participation. Participation was restricted to individuals who had no previous training in art or the history of art, and had normal or corrected-to-normal vision without color blindness. All participants gave written informed consent, and the study was approved by the Academic Committee of the Department of Psychology at South China Normal University.

#### Materials

Seventy-two traditional Chinese landscape paintings and seventy-two realistic oil landscape paintings were selected from the archives of http://www.artcyclopedia.com and http://www.namoc.org. The contents of the landscape paintings mainly included sky, mountains, rivers, trees, flowers, meadows, houses, and boats. To avoid brain activation associated with viewing faces, artworks that contained close views of humans were discarded. The paintings were chosen from a variety of historical periods (from the 13th century to the recent past). Stimuli were resized to fit within a 500 × 500 pixels frame. A prior esthetic rating experiment confirmed that there was no significant difference between traditional Chinese landscape paintings and realistic oil landscape paintings in average esthetic preference levels, *t*(25) = 0.51, *p* > 0.05.

#### Procedures

During the experiment, participants were asked to view each painting for as long as they liked (self-paced), and then answer five questions on a rating scale ranging from 1 (not at all) to 5 (to a great extent). The questions were as follows:

“1. I liked this painting.

2. I felt absorbed by this painting.

3. I felt calm while viewing this painting.

4. My mind was wandering while viewing this painting.

5. I recognized many objects in this painting.”

The above five questions are associated with the following mental processes, respectively: preference, object-related absorption^1^, relaxation, mind wandering, and object recognition.

### RESULTS

As shown in **Table 1**, preference levels were not significantly different between traditional Chinese landscape paintings and realistic oil landscape paintings. However, traditional Chinese landscape paintings had significantly higher ratings in relaxation [*t*(27) = 4.22, *p* < 0.05] and mind wandering [*t*(27) = 3.33, *p* < 0.05], but lower ratings in absorption [*t*(27) = -4.24, *p* < 0.05] and object recognition [*t*(27) = -6.47, *p* < 0.05], compared with realistic oil landscape paintings (**Table 1**). These results suggest that while viewing traditional Chinese landscape paintings, viewers became more inward oriented (i.e., experienced greater levels of relaxation and mind wandering). However, when the same viewers were shown the realistic oil landscape paintings, they became more externally oriented toward features of the work, potentially attempting to identify the objects and narrative contained within the artwork. The subjective differences between experiencing the different types of artwork were consistent with our predictions, and serve to validate the use of our stimulus set for the fMRI paradigm in Experiment 2.

**Table 1:** The mental processes during landscape painting appreciation in Experiment 1.

	Traditional Chinese landscape painting		Realistic oil landscape painting		*t*-value (*n* = 28)
	*M*	SD	*M*	SD	
Viewing Time (ms)	3631.21	2132.36	3623.21	2432.26	0.04
Preference	3.49	0.46	3.40	0.57	1.19
Absorption	2.56	0.59	3.06	0.53	-4.24^∗^
Relaxation	3.23	0.52	2.69	0.56	4.22^∗^
Mind Wandering	2.94	0.51	2.61	0.57	3.33^∗^
Object Recognition	2.69	0.54	3.03	0.49	-6.47^∗^

*Refers to the *t*-value was significant at *p* < 0.05.

## EXPERIMENT 2: fMRI EXPERIMENT

In Experiment 2, we used fMRI to explore the neural substrates of the effect of viewing traditional Chinese landscape paintings on a task requiring cognitive control (i.e., the flanker task). Specifically, we examined whether the phenomenological experience of the calming “after-effect” produced by viewing traditional Chinese landscape paintings would produce activity in brain areas known to be involved in tasks requiring cognitive control (Cahill, 1960; Zong, 2007; Law, 2011).

### METHOD

#### Participants

Twenty (11 female, 9 male) right-handed, Chinese college-aged participants from Southwest University in Chongqing, China were recruited and paid for their participation. None of them had participated in Experiment 1. Participation was restricted to individuals who had no previous training in art or the history of art, and had normal or corrected-to-normal vision without color blindness. All participants gave written informed consent, and the study was approved by the Academic Committee of the Department of Psychology at South China Normal University.

#### Materials

**FIGURE 1:**
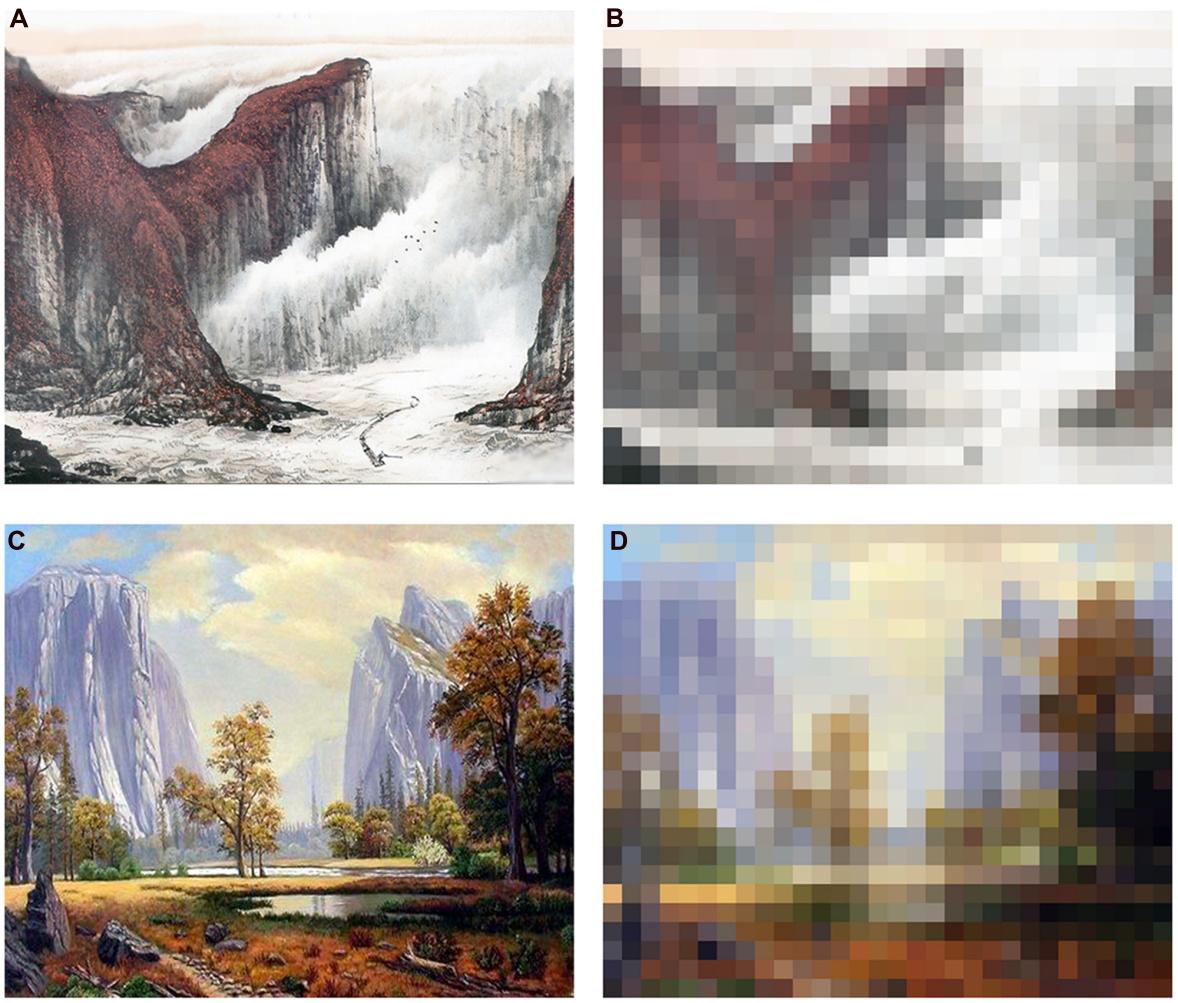
**Examples of the four types of paintings used. (A)** An example of an original traditional Chinese landscape painting; **(B)** An example of a scrambled traditional Chinese landscape painting; **(C)** An example of an original realistic oil landscape painting; **(D)** An example of a scrambled realistic oil landscape painting.

***Artworks*.** The paintings were identical to those used in Experiment 1. The original paintings are referred to as either Chinese landscape paintings (CO) or realistic oil landscape paintings (RO). A scrambled version for each original painting was created in Adobe Photoshop CS4 (see Vartanian and Goel, 2004). The paintings that were created using this process are referred to as scrambled traditional Chinese landscape paintings (CS) and scrambled realistic oil landscape paintings (RS). Scrambled paintings served as a comparison condition because they retained the overall colors and general composition of the original paintings while lacking perceptual detail. Thus, four types of paintings were developed (see **Figure 1**).

***Flankers*.** Two hundred and 88 flanker trials were presented. During each trial, five arrows that pointed to the left (<) or right (>) were displayed in the center of a rear projection screen. Both congruent and incongruent flankers were presented. For the congruent flankers, the central arrow and the side arrows pointed in the same direction (e.g., >>>>> or <<<<<). For the incongruent flankers, the side arrows pointed in the opposite direction of the central arrow (e.g., >><>> or <<><<). Both congruent and incongruent flankers included 144 trials each, of which 36 were presented following original traditional Chinese landscape paintings (18 congruent, or COc and 18 incongruent, or COi), 36 following scrambled traditional Chinese landscape paintings (18 CSc and 18 CSi), 36 following original realistic oil landscape paintings (18 ROc and 18 ROi), and 36 following scrambled realistic oil landscape paintings (18 RSc and 18 RSi). Thus, in total eight types of flankers trials were constructed and were included in the general linear model (GLM; see Data Analysis).

#### Procedures

In the scanner, trials were presented in an event-related design. For painting *viewing*, there was one variable: painting type (CO, CS, RO, and RS). For the flanker task, there were two variables: painting type (CO, CS, RO, and RS) and Congruency (congruent and incongruent). [Note that, because of the timing of our event-related trials, neural responses for viewing the painting and the flanker tasks purposefully overlapped; their behavioral responses could of course be examined in isolation] Each painting was presented for 3.5 s and was followed by a fixation of 0.5 s. Participants were instructed to view the painting in a subjective and engaged manner, experiencing the feelings it evoked, and to judge whether they liked the painting or not by pressing either the ‘like’ or ‘dislike’ key. They gave their like/dislike rating during the 3.5 s while the artwork was on the screen. Key responses were counterbalanced between participants. When the fixation cue was presented, participants were instructed to attend to the central fixation cue. Next, a flanker trial was presented for 1 s. Participants were instructed to press the left key if the center arrow was pointing to the left (<) and the right key if the center stimulus was pointing to the right (>). Trials were separated by a variable inter-trial-interval (ITI) of 1.5—9 s. After scanning, participants were instructed to rate the familiarity, complexity, and beauty levels of the paintings ranging from 1 (very unfamiliar, very simple, and very ugly) to 4 (very familiar, very complex, and very beautiful). These ratings were done to ensure that the Chinese landscape paintings and realistic oil landscape were perceptually matched on familiarity, complexity, and beauty level across participants. Each participant took part in two functional scans. Each scan lasted about 12 min.

#### Image acquisition

Whole-brain functional data were acquired on a 3T Siemens Trio scanner in 40 axial slices (3 mm × 3 mm × 3 mm voxels) parallel to the anterior commissure- posterior commissure (AC-PC) line with a T2^∗^-weighted spiral in-out sequence developed by Dr. Gary Glover [repetition time (TR) = 2000 ms, echo time (TE) = 30 ms, flip angle = 90°, field of view (FOV) = 192 mm]. Structural data were acquired with a T1-weighted spoiled gradient-recalled sequence (1 mm × 1 mm × 1 mm; TR = 19 ms, TE = 5 ms, flip angle = 20°).

#### Data analysis

Functional images were preprocessed using SPM8. For each participant, all functional scan volumes were corrected for motion artifacts. The mean image was then co-registered to the high-resolution T1-weighted image. The estimated transformation parameter was applied to individuals to normalize all scan volumes to the stereotactic MNI space. The normalized scans were smoothed using an 8-mm full-width at the full-width half-maximum (FWHM) Gaussian kernel.

**FIGURE 2:**
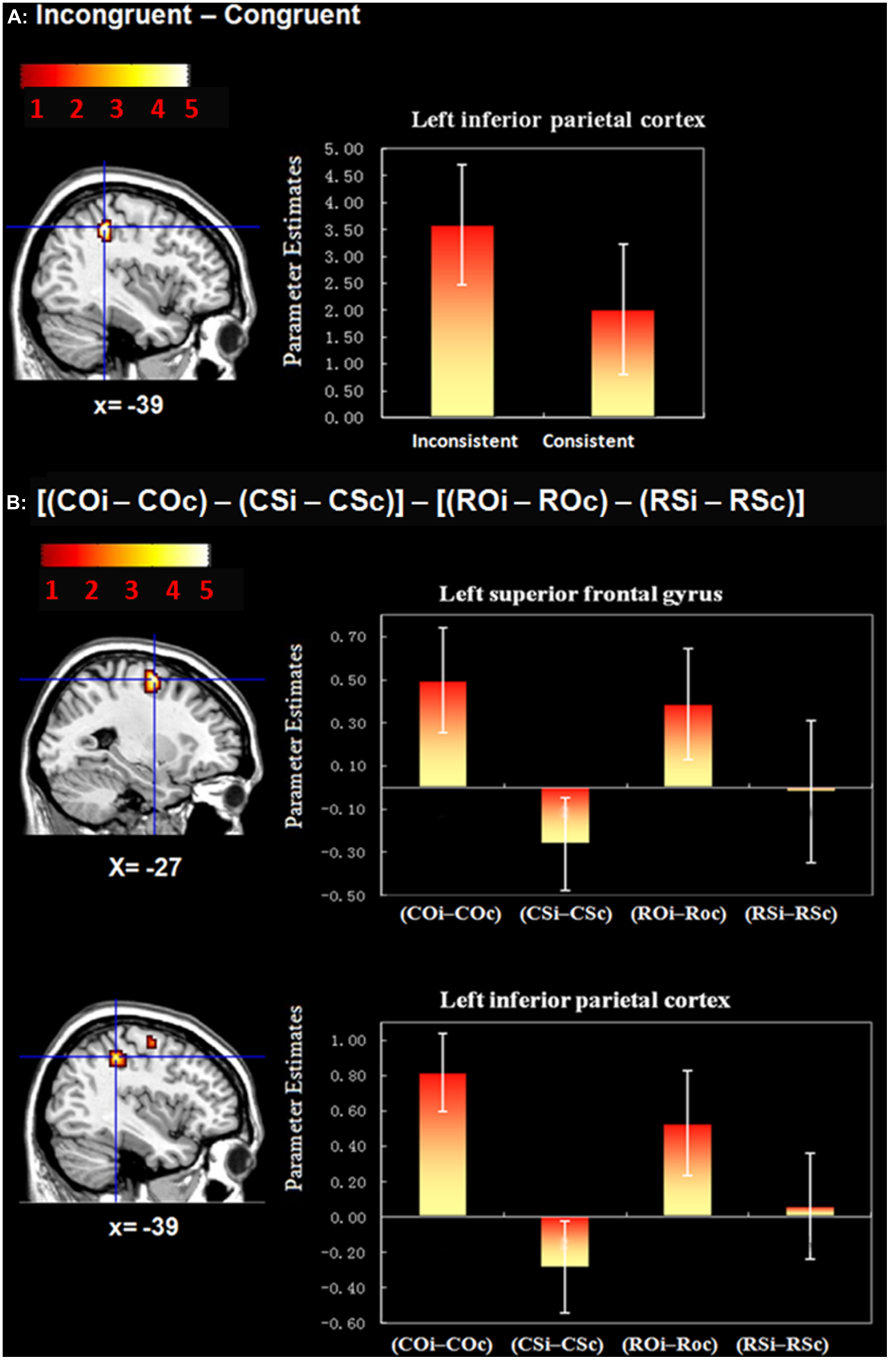
**Whole brain activation during the flanker task. (A)** Activation of the contrast “Incongruent–Congruent.” The left inferior parietal cortex (IPC) was found to exhibit greater activation; **(B)** Activation of the contrast “[(COi–COc) – (CSi–CSc)] – [(ROi–ROc) – (RSi–RSc)].” The left superior frontal gyrus (SFG) and IPC were found to exhibit greater activation in this contrast. Mean parameter estimates in each brain area for each contrastare shown. By using a leave-one-out-cross-validation (LOOCV) analysis (Kriegeskorte et al., 2009), the data used to define an regions of interest (ROI) and the data extracted from this ROI (activation in the bar graphs) are independent.

In the single-participant GLM, events were modeled using a canonical HRF and its temporal derivative. Multiple regressors were used in order to overcome the potential slice-timing problem. According to previous research, the canonical HRF and its temporal derivative can efficiently detect differences in the latency of blood oxygenation level-dependent (BOLD) responses to brief events (Friston et al., 1998; Henson et al., 1999, 2002). Eight conditions were included in the model: COc, COi, CSc, CSi, ROc, ROi, RSc, and RSi. The GLM also included covariates of no interest (session mean, motor response, false response, and their respective temporal derivatives). The reaction times (RTs) of the eight conditions in the flanker task (COc, COi, CSc, CSi, ROc, ROi, RSc, and RSi) were also included in the GLM as a first-order parametric modulator. The onset time of COc, COi, CSc, CSi, ROc, ROi, RSc, and RSi trials were locked to the onset of the flanker task (and thus contained activation from the painting appreciation task encountered 0.5 s earlier). At the first level, analyses were performed individually for each participant and contrast images were subsequently entered into a second-level analysis treating participants as a random factor. At the group level, analyses were conducted at a voxel-wise threshold of *p* < 0.001 (uncorrected for multiple comparisons), and a cluster threshold of *p* < 0.05 (corrected for multiple comparisons; Forman et al., 1995; Lieberman and Cunningham, 2009). The activation in the regions of interest (ROI) was plotted by a leave-one-out-cross-validation (LOOCV) analysis (Kriegeskorte et al., 2009). Each ROI (i.e., the IPC and the SFG) was defined using a sphere with 10-mm radius centered on the peak voxel of the cluster. As a result of the LOOCV analysis, the data used to define an ROI and the data extracted from this ROI (bar graphs in **Figure 2**) are independent.

### RESULTS

#### Behavioral data

***Online behavioral data*.** We performed separate repeated-measures ANOVAs for the preference ratings and RTs in the painting-viewing task (**Table 2**) as well as the accuracy and RTs in the flanker task (**Table 3**).

**Table 2:** Behavioral results of the painting-appreciation task in Experiment 2.

Painting appreciation	RTs (ms)		Ratings	
	*M*	SD	*M*	SD
**CO**	971.20	241.43	1.45	0.61
**CS**	779.60	117.40	3.80	0.56
**RO**	937.62	280.93	1.31	0.40
**RS**	815.72	158.20	3.69	0.67

*In the scanner, the “like” response was recorded as “1” and the “dislike” response was recorded as “4.”*

**Table 3:** Behavioral results of the flanker task in Experiment 2.

Flanker task	RTs (ms)		Accuracy rates (%)		
	*M*	SD	*M*	SD
Congruent	510.85	50.39	0.98	0.03	
Incongruent	570.45	66.73	0.96	0.05	
COc	498.12	53.61	0.97	0.04	
COi	550.17	55.55	0.96	0.04	
CSc	508.87	56.96	0.98	0.04	
CSi	553.78	62.51	0.96	0.04	
ROc	499.15	51.90	0.99	0.03	
ROi	547.96	68.25	0.95	0.06	
RSc	503.84	46.90	0.99	0.02	
RSi	547.57	69.56	0.94	0.08	

***Painting viewing*.** For the preference ratings of the painting-viewing task, there was a significant main effect for painting type, *F*(3,57) = 37.69, *p* < 0.05. Planned *t*-tests showed that participants preferred CO and RO significantly more than CS and RS (*p* < 0.05). However, there were no significant differences between CO and RO (*p* > 0.05), or CS and RS (*p* > 0.05). These results show that participants preferred the original paintings, Chinese or oil landscapes, more than the scrambled images. Note that the two stylistic variations on landscape paintings were liked equally—replicating the results of Experiment 1. For the RTs of painting viewing, the main effect of painting type was significant, *F*(3,57) = 8.57, *p* < 0.05. Planned *t*-tests showed that participants responded significantly faster in CS and RS than CO and RO (*p* < 0.05 after correcting for multiple comparisons using the Tukey procedure). It is not surprising that rating scrambled images required less time than rating landscape paintings because the latter are more likely to activate processes associated with recognition, reflection and awareness (compared to processing randomly scrambled images).

***Flanker task*.** The overall accuracy of the flanker task was 97% (SD = 5%). The main effect of congruency was significant, *F*(1,19) = 17.88, *p* < 0.05. Subsequent *post hoc* comparisons showed that participants were more accurate on congruent than incongruent trials (*p* < 0.05). However, contrary to our prediction, there was no main effect of painting type, or a painting type × congruency interaction (see **Table 3**). For the RTs in the flanker task, the main effect of congruency was significant, *F*(1,19) = 68.5, *p* < 0.05. Planned *t*-tests showed that participants responded significantly faster in the congruent than the incongruent condition (*p* < 0.05). It is worth reiterating that the RTs of each condition in the flanker task were included in the GLM of the fMRI data as a first-order parametric modulator. The RT results of the flanker task are consistent with the idea that coherent flanker images possess the quality of “good gestalt” which increases accuracy and speeds up judgment time (Köhler, 1929). Importantly, contrary to our prediction, there was no main effect of painting type or a painting type × congruency interaction (see **Table 3**).

***Post-scan behavioral data*.** Paired *t*-tests comparing Chinese landscape paintings and realistic oil landscape paintings did not show a significant difference in complexity [*t*(19) = -0.48, *p* > 0.05], familiarity [*t*(19) = 0.238, *p* > 0.05], or beauty ratings [*t*(19) = -1.14, *p* > 0.05]. This is likely because complexity, familiarity, and beauty are important qualities in both kinds of artworks (Berlyne and Ogilvie, 1974). In addition, these post-scan ratings are broadly consistent with the findings of Experiment 1, which showed no difference in preference.

One might be inclined to expect that Chinese participants should be more familiar with the traditional Chinese landscape paintings than realistic oil paintings. However, in order to make the stimuli comparable, the paintings used in the current study were chosen to be unfamiliar, and the two types of paintings were matched in terms of both content and motif. Furthermore, the participants had no previous training in art. Therefore, the familiarity of the two sets of paintings did not differ, and this was confirmed by the behavioral ratings of the participants. This non-significant result excluded the possibility of the confounding effects of familiarity on brain activation.

#### fMRI data

Given that RTs were modeled in our analysis of fMRI data, we can rule out linear effects of this confounding variable explaining the imaging results reported below.

***Brain activity associated with cognitive control during the flanker task*.** We began our analysis by conducting a manipulation check, which involved comparing brain activity between incongruent and congruent flanker trials (collapsed across painting type). Our aim was to identify brain regions associated with cognitive control. This analysis revealed significant activation in the left IPC (**Table 4**; **Figure 2**). This result is consistent with previous studies (Casey et al., 2000; Hazeltine et al., 2000; Durston et al., 2003), and suggests that the flanker task adopted in the current study was successful in eliciting activation in brain regions related to cognitive control.

**Table 4:** Peak voxel coordinates and cluster size for all regions obtained from the whole brain random-effects analysis in Experiment 2 (*p* < 0.001 uncorrected, *p* < 0.05 corrected at the cluster level).

Anatomical region	Hemisphere	x	y	z	*T*-Score	Cluster size
**Incongruent flankers – Congruent flankers**
Inferior parietal cortex (IPC)	L	-39	-37	46	5.43	61
**[(COi–COc) – (CSi–CSc)] – [(ROi–ROc) – (RSi–RSc)]**
Superior frontal gyrus (SFG)	L	-27	-4	61	6.19	118
Inferior parietal cortex (IPC)	L	-39	-37	46	5.59	66

**T*-scores computed by statistical parametric mapping quantified the statistical difference between the two conditions. Coordinates refer to the MNI stereotactic space*.

***Stronger cognitive control during the incongruent flanker trials after appreciating traditional Chinese landscape paintings*.** To explore whether participants experienced stronger cognitive control during incongruent flanker trials after appreciating traditional Chinese landscape paintings, we performed the contrast of “[(COi–COc) – (CSi–CSc)] – [(ROi–ROc) – (RSi–RSc)].” Results revealed activation in the left IPC and left SFG. Scrambled versions of each painting style were subtracted from the original versions to ensure that any activation observed reflected high-level cognitive differences as a result of viewing incongruent compared with congruent trials, and not simply low-level visual differences between the paintings (e.g., color, spatial frequency, etc.). The observation of relatively greater activations in these regions is consistent with the idea that more cognitive and attentional resources were recruited on incongruent compared with congruent trials following exposure to traditional Chinese landscape paintings than realistic oil and/or scrambled images. Indeed, this interpretation is bolstered by the involvement of IPC and SFG in attention and WM, respectively (du Boisgueheneuc et al., 2006; Toro et al., 2008). The reverse contrast “[(ROi–ROc) – (RSi–RSc)] – [(COi–COc) – (CSi–CSc)]”did not reveal any significant areas of activation, indicating that our results are specific to the incongruent trials when viewing traditional Chinese landscape paintings.

## DISCUSSION

Experiencing art is a multifaceted phenomenon, and not merely reflected by the experience of pleasure and reward. The calming effect of viewing certain artworks, like Chinese landscape paintings, as well as listening to certain forms of music, has long been recognized both phenomenologically and theoretically (Cahill, 1960; Zong, 2007; Law, 2011). The present study was conducted to investigate if the calming effect induced by viewing traditional Chinese landscape paintings would make disengagement from that mental state more difficult, as measured by performance on a cognitive control task. In accordance with our *a priori* hypothesis, the results of Experiment 1 demonstrated that compared with realistic oil landscape paintings, traditional Chinese landscape paintings were associated with higher subjective ratings of relaxation and mind wandering, but lower ratings of object-oriented absorption and recognition. That is to say, when appreciating realistic oil landscape paintings, people might experience an attention-focusing mental state, during which they might pay more attention to the details of the painting content *per se* (e.g., color, shape, and objects). However, when appreciating traditional Chinese landscape paintings, people might experience a relatively greater mind-wandering mental state, during which they might become relaxed and tend to engage in mental states possibly unrelated to the painting content *per se* (e.g., imagining, recalling, and thinking about potentially unrelated information; Smallwood et al., 2003; Smallwood and Schooler, 2006; Mason et al., 2007; Vessel et al., 2012).”

Because appreciators experience mental travel and tend to lose themselves in traditional Chinese landscape painting, one would expect this orientation to induce a calming “after-effect” phenomenon in the form of difficulty to disengage from the painting. In other words, viewing certain artworks may inhibit the immediate shift from art appreciation to other, especially attention-demanding, cognitive tasks. However, the behavioral results of Experiment 2 did not support this prediction. Specifically, we did not observe that viewing traditional Chinese landscape paintings compared with realistic oil landscape paintings impaired performance on the flanker task. In combination, our behavioral results suggest that although viewing traditional Chinese landscape paintings effectively induces states of relaxation and mind wandering (Experiment 1), disengagement from that state in the service of an attentionally – and cognitively demanding task might not be any more difficult than disengaging from the mental state associated with object-oriented perception invoked by viewing realistic oil landscape paintings.

Importantly, however, despite the absence of a behavioral effect of painting type on the flanker task, we did observe greater activation for incongruent compared with congruent trials in the SFG and IPC after viewing traditional Chinese landscape paintings as opposed to realistic oil landscape paintings. Much evidence links activation in IPC and SFG to attention and WM, respectively. For example, a large-scale meta-analysis of functional imaging studies isolated the IPC as an important hub in the fronto-parietal attention network (Toro et al., 2008). In addition, neuropsychological studies of patients with focal brain lesions have shown the necessary role that left SFG plays in WM (du Boisgueheneuc et al., 2006), complementing evidence from functional imaging studies. Our neural results suggest that although disengagement from the mental state associated with viewing traditional Chinese landscape paintings was not associated with greater levels of cognitive control, this switch might have nevertheless placed greater demands on the brain’s attention and WM networks compared to the switch associated with viewing realistic oil landscape paintings.

Another possibility for our failure to detect a behavioral effect as a function of painting type on the flanker task might have been a ceiling effect on the latter, where overall accuracy was 97%. In other words, the flanker task might have been too easy, and therefore not sensitive enough to measure the difference between different kinds of paintings. It is possible that, had the behavioral flanker task been harder, we might have observed a behavioral effect. Importantly, oftentimes behavioral and neural data do not precisely mirror each other, but this does not invalidate one set of data as compared with the other (see Xu et al., 2007, for a good example of a dissociation between behavioral and fMRI findings, as related to task difficulty). Future studies involving a more difficult cognitive control task will be needed to test this possibility. One possibility is to use a modified version of the flanker task, where the perceptual features of the central target and flanking distracters are more difficult to discriminate. Another possibility is to use a Stroop task (Stroop, 1935), or a Navon-type task (Navon, 1977) where global and local features of compound stimuli are discriminated. Both of those tasks contain congruent and incongruent trials that have clear differences in behavioral performance (definitely in RT, perhaps also in accuracy). Finally, while we think it is unlikely that the null results in the behavioral measures can be explained by the fact that participants had to perform a like/dislike task before engaging in the flanker task, future research should examine if there are differences in behavioral performance on the flanker task when using single versus dual-task paradigms.

To date, studies in neuroesthetics have by and large focused on uncovering the neural correlates of viewing artworks. This study makes an important contribution to the field by examining how engagement with artworks affects subsequent cognitive performance. Thus, this work may ultimately improve our understanding of the ways in which esthetic engagement affects our future decisions (Lebreton et al., 2009; Kirk et al., 2011).

## CONCLUSION

Our results are consistent with the idea that viewing traditional Chinese landscape paintings facilitates relaxation and mind wandering (Experiment 1). However, contrary to our prediction, behavioral data demonstrated that disengagement from this mental state does not place greater demands on cognitive control, as measured by the flanker task (Experiment 2). Rather, the neural data—in the form of greater activations observed in IPC and SFG for incongruent compared with congruent flanker trials—suggest that the brain’s attention and WM systems might be differentially activated when switching from traditional Chinese landscape paintings compared with realistic oil landscape paintings. Building on the present results, this possibility can be tested directly in future studies.

## Conflict of Interest Statement

The authors declare that the research was conducted in the absence of any commercial or financial relationships that could be construed as a potential conflict of interest.

## Footnotes

1 In this context absorption has been defined as “object-oriented” to distinguish it from another connotation of the term, which involves “a disposition, penchant, or readiness to enter states characterized by marked cognitive restructuring” or a “readiness to depart from more everyday life cognitive maps and to restructure … one’s representation of one’s life and its boundaries” (Glisky et al., 1991). In other words, in the present context absorption meant added focus on the depicted scene rather than an internally oriented cognitive stance.
